# Palliative radiotherapy for gastric cancer: a systematic review and meta-analysis

**DOI:** 10.18632/oncotarget.15554

**Published:** 2017-02-20

**Authors:** Jeremy Tey, Yu Yang Soon, Wee Yao Koh, Cheng Nang Leong, Bok Ai Choo, Francis Ho, Balamurugan Vellayappan, Keith Lim, Ivan WK Tham

**Affiliations:** ^1^ Department of Radiation Oncology, National University Hospital, National Cancer Institute of Singapore, Singapore

**Keywords:** radiotherapy, gastric cancer, palliation, bleeding, pain

## Abstract

**Background/Purpose:**

To review the efficacy and toxicity of palliative radiotherapy (RT) for symptomatic locally advanced gastric cancer (GC) and to determine the optimal RT schedule for symptom palliation.

**Methods:**

We searched MEDLINE and CENTRAL for eligible studies published from 1995 to 2015. Outcomes of interest were relief of bleeding, pain and obstruction.

**RESULTS:**

Seven non-comparative observational studies were included. There were large variations in RT dose and fractionation. The pooled overall response rates for bleeding, pain and obstruction symptoms were 74%, 67% and 68% respectively. There was no difference in response rate of bleeding between regimens with high biological equivalent dose (BED) of = 39Gy versus regimens with low BED<39Gy regimens (p value =0.39). Grade 3 to 4 toxicities occurred in up to 15% of patients for patients treated with RT alone and up to 25% of patients treated with chemoradiotherapy. Health-related quality of life (HRQL) outcomes were not reported.

**Conclusion:**

More than two-thirds of patients receiving RT would have a clinical benefit. Low BED regimens appear to be adequate for symptom palliation. Toxicity rates appear acceptable for patients treated with RT alone. The optimal dose fractionation regimen for symptom palliation remains unclear. Prospective studies to determine the effects of palliative gastric RT on HRQL outcomes are warranted.

## INTRODUCTION

Local tumour progression is often a cause of the presenting symptoms in patients with recurrent and locally advanced primary gastric cancers. Even in GC patients that do not initially present with local symptoms, they eventually may require intervention for progressive local symptoms while on palliative chemotherapy.

Common local symptoms that patients present with or develop include obstruction, bleeding or pain. The intervention options for relieving local symptoms include palliative radiotherapy (RT), palliative chemotherapy, gastric bypass surgery, palliative gastrectomy, and endoscopic stenting. The ideal treatment modality should be effective yet having minimal side effects. RT is a non invasive treatment for these local symptoms.[1-4] Interestingly, there is very scarce literature with regards to the tolerability and the efficacy of RT in palliating the local symptoms. Of debate is also the optimal dose fractionation regimen for effective symptom palliation. In patients with advanced disease, hypofractionated regimens have been increasingly used. Small case series have reported efficacy with hypofractionated regimens, with the added advantage of reduced overall treatment time.[5] Others have suggested that low BED (Biologically Effective Dose) regimens may not be adequate for palliation of obstruction and may be associated with poorer local control compared to higher BED regimens. [6] To date, there have been no published reviews summarizing the benefits of palliative RT for palliation of local symptoms.

The aim of this study is to determine the effectiveness and toxicities of RT in the palliation of local GC symptoms. We also sought to determine the optimal dose fractionation regimen by evaluating the different treatment schedules used in symptom palliation.

## RESULTS

### Search results

**Figure 1:**
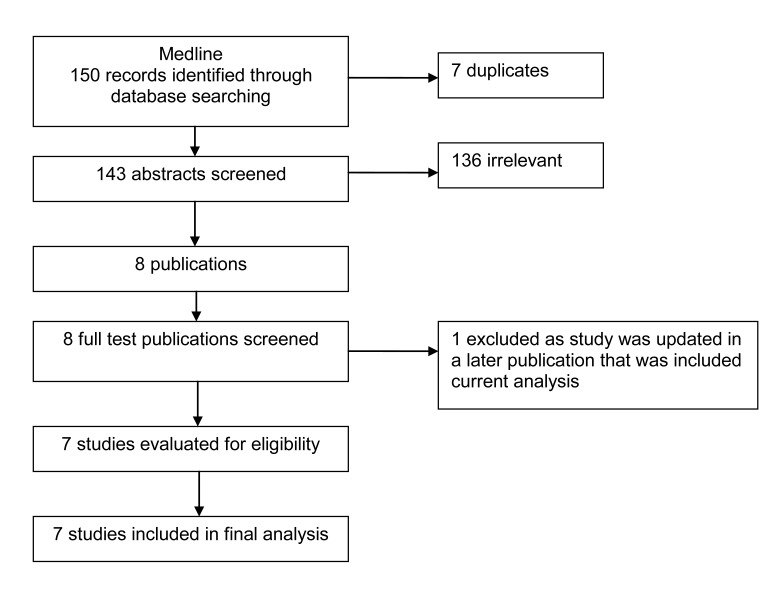
Study flow chart

Figure 1 details the search strategy. We found seven non-comparative observational studies including 291 patients who received palliative RT for relief of local symptoms.[5, 6, 13-16]

**Figure 2:**
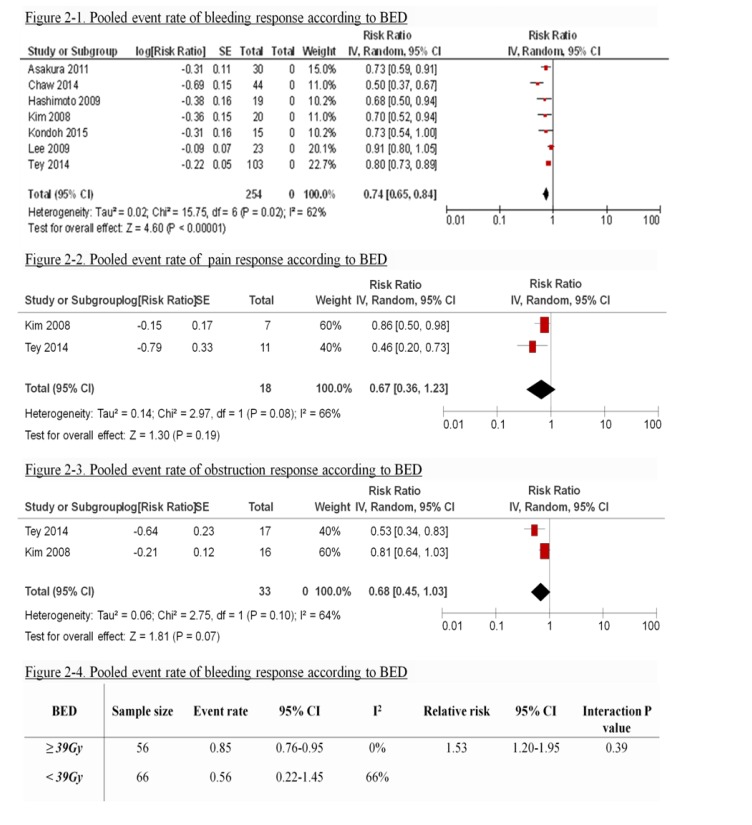
Pooled event responcse according to index symptom

### Patient characteristics and symptoms

The characteristics of the seven studies are summarised in Table 1. The studies were published from 2008 to 2015. All were retrospective reviews. The sample size of the included studies ranged from 15 to115 patients. Seventy percent (206/291) of patients were male. Median age of included patients was 66 years (range 61-78 years). Median follow-up ranged from 2.1 to 35.4 months. All patients underwent gastroscopy which confirmed gastric bleeding.

**Table 1:** Characteristics of studies of palliative radiotherapy for gastric cancer

Author/ Year	Study design and treatment period	Patients		Computed Tomography (CT) planned	Radiotherapy (dose/fraction size/treatment period	Relevant outcome	Patient follow-up (months)
		Number	Stage				
Kim MM 2008^6^	Retrospective 1996-2004	37	mixed	NR	Median 35Gy (25-36Gy)	Requirement for further intervention or presence of symptoms at follow-up	3.1
Hashimoto 2009^12^	Retrospective 1994-2007	19	metastatic	Yes	Median 40Gy (2-50Gy in 1.8-3Gy / Fr)	Requirement for blood transfusions after RT	NR
Lee JA 2009^13^	Retrospective 2002-2007	23	mixed	No	30 Gy / 10 fr	Changes in HB, number of transfusions before and after RT	4
Asakura 2011^14^	Retrospective 2002-2007	30	mixed	Yes	30 Gy / 10 fr	Requirement for blood transfusions after RT	3.5 (0.5-19.6)
Chaw 2014^5^	retrospective	52	mixed	NR	8Gy single fr 20Gy / 5 fr	Changes in HB, number of transfusions before and after RT	NR
Tey 2014^15^	Retrospective 1999-2012	115	mixed	Yes (93%)	8-40Gy	Symptom response 3 point scale	2.8
Kondoh 2015^16^	Retrospective 2007-2012	15	metastatic	Yes	Median 30Gy 30-40Gy/10-20 fr	Response if : No evidence of bleeding, increase in HB, no interventions for bleeding, for seven days	35.4 (0.9-82)

Majority of patients had adenocarcinoma of the stomach. Two studies included one patient with squamous cell carcinoma [5, 12] and one study included one patient with carcinoma with neuroendocrine features of the stomach. [5]

All patients received RT for palliation of local symptoms. Five studies included patients treated with chemoradiotherapy.[ 6, 12-14, 16] All patients in the studies had bleeding as the index symptom. Two studies included patients who presented with pain and obstruction.[6, 15] Five studies included patients with both locally advanced and metastatic disease. [5, 6, 13-15] Two studies included patient with metastatic disease only. [12, 16]

### Methological quality of included studies

**Figure 3:**
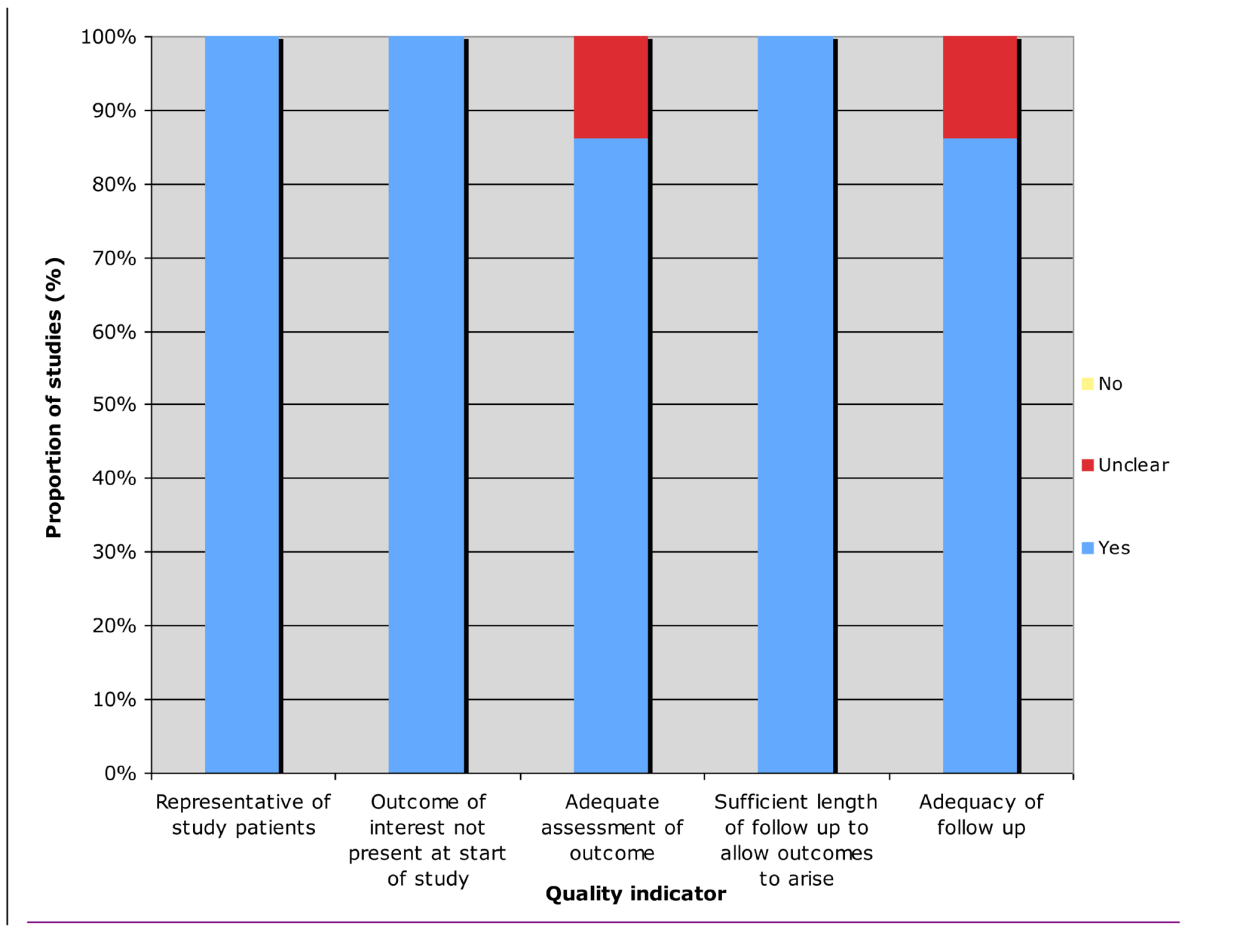
Assessment of quality of included studies

The methodological quality of included studies in summarized in Figure 3. All studies enrolled a representative sample of patients and defined the outcomes of interest at the start of the studies. Only one study did not provide adequate assessment of outcome. ^6^ Kim et al did not provide objective measures of response in their study. All studies allowed for sufficient length of follow up to allow outcomes to arise. Only one study did not clearly account whether all included patients were being followed up.^6^

### Radiotherapy dose, fractionation and target and technique

There were wide variations in dose fractionation regimens between studies. Fraction sizes ranged from 1.8 to 8 Gy and total doses ranged from 8Gy to 50Gy. The most common dose fraction regimen used was 30Gy in 10#. One paper did not state the distribution of dose/fractionation regimen.[13] 20% (57/291) of patients were treated with chemoradiotherapy. Four of the studies planned patients using 3 dimensional simulation [12, 14-16], one used 2-dimensional simulation [13] and two studies did not report the planning technique used.[5-6] Target volumes definitions for RT were variable, including the whole stomach or partial stomach, with or without regional lymph nodes. Only one study provided dose constraints used for RT planning. [15] The most common field arrangement was Anterior-Posterior/ Posterior-Anterior fields.

### Treatment response

Response criteria varied across studies for bleeding, pain and obstruction. Different time points were used to assess symptom response. 4 studies evaluated for symptom response one month after RT [5, 12, 14-15], one study on the day of completion of RT [13], one study after 7 days of starting RT [16] and one study ‘at follow-up’ after RT.[6]

### Bleeding

All 7 studies included a total number of 254 patients who presented with gastric bleeding. Varying definitions were used to define treatment response. (Table 1) Response to RT ranged from 50% to 80.6%. The median duration of response ranged from 1.5 to 11.4 months. Symptom response to palliative RT is seen in Table 2.

**Table 2:** Symptomatic response to palliative radiotherapy for gastric cancer

Author/ year	Radiotherapy (dose/fraction size/treatment period	Index symptom	Response	Survival (months)	Duration (months)	BED correlation
Kim MM 2008^6^	Median 35Gy (25-36Gy), 24/37 patients had concurrent chemotherapy)	Gastric bleeding, pain, obstruction	Bleeding 70%(14/20), pain 86% (6/7), obstruction 81% (13/16)	5.2	Bleeding (11.4), pain (NR), obstruction (6.2)	BED 41 Gy10 conferred better local control of symptoms but not overall survival
Hashimoto 2009^12^	Median 40Gy (2-50Gy in 1.8-3Gy / fr) (4/19 patients had CRT)	Gastric bleeding	Bleeding 68% (13/19)	3.4	1.5	BED 50 Gy10 conferred higher success rate of haemostasis compared to those received BED <50 Gy10
Lee JA 2009^13^	30 Gy / 10 fr (12/30 patients had CRT)	Gastric bleeding	Bleeding 91% (21/23)	4	4	NR
Asakura 2011^14^	30 Gy / 10 fr (12/30 patients had concurrent chemotherapy)	Gastric bleeding	Bleeding 73% (22/30)	3.6	3.3	BED 39 Gy10, conferred a response rate comparable to higher BED (50 Gy10 or more)
Chaw 2014^5^	8Gy single fr 20Gy / 5 fr	Gastric bleeding	Bleeding 50% (22/44, 8 patients unevaluable)	5.3	NR	BED 28 Gy10, conferred a similar response rate compared to other studies
Tey 2014^15^	8-40Gy	Bleeding, pain, obstruction	Bleeding 80.6%(83/103), pain 45.5% (5/11), obstruction 51.2% (9/17)	2.8	Bleeding (3.3), pain (7.8), obstruction (3.2)	Trend for poorer local control for BED ≤39 Gy10
Kondoh 2015^16^	Median 30Gy (30-40Gy in 2-3Gy / fr) (5/15 patients had CRT)	Bleeding	Bleeding 80.6%(11/15-RT:7/15 CRT: 4/15)	2.1	NR	NR

Only 122 patients were eligible for subgroup analyses as BED data were available. The overall pooled response rate for bleeding is 74% (95% CI 0.64-0.85. I^2^ = 68%) (Figure 2-1). There was no difference in response rate of bleeding between regimens with biological equivalent dose (BED) of ≥ 39Gy versus regimens with BED<39Gy (relative risk 1.53, 95%CI 1.20-1.95; p value =0.39) (Figure 2-4). Two studies suggested that local control was inferior for patients treated with BED regimens of <41Gy. [6, 15]

### Pain

Two studies included a total of 18 patients who presented with pain. [6, 15] One study defined response as patient not requiring interventions such as neurolysis on follow-up. [6] The other study used a 3 point scale to grade symptom response. [15] Response rates for pain ranged from 45.5% to 86% (Table 2). Median duration of response for pain was 7.8 months reported in one study. [15] The overall pooled response rate for pain is 67% (95% CI 0.36 -1.23. I^2^ =66%) (Figure 2-2).

### Obstruction

Two studies included a total of 33 patients who presented with obstruction.[6, 15] One study defined response as patient not requiring interventions such as stenting on follow-up.[6] The other study used a 3 point scale to grade symptom response.[15] Response rates for obstruction ranged from 51.2% to 81% (Table 2). Median duration of response for obstruction ranged from 3.2 to 6.2 months. The overall pooled response rate for obstruction is 68% (95% CI 0.45-1.03. I^2^ =64%). (Figure 2-3)

### Toxicity

An overview of the toxicities reported in all studies is presented in Table 3. One study did not report toxicity outcomes.[5] Validated grading scales such as Radiation Therapy Oncology Group (RTOG) or Common Toxicity Criteria (CTC) scales were used. Chemoradiotherapy was associated with increased toxicities. Grade 3 to 4 acute toxicities occurred in up to 15% of patients for patients treated with RT alone and up to 25% of patients treated with chemoradiotherapy. One study reported late grade 3 Gastrointestinal hemorrhage in 8% (1/12) patients who underwent chemoradiotherapy.

**Table 3:** Toxicity reported in studies of palliative radiotherapy for gastric cancer

Author/ year	Acute toxicity (CTC or RTOG)			Late Toxicity
	Gastrointestinal (Grade >3)	Skin/connective tissue (Grade >3)	Others (Grade >3)	
Kim MM 2008^6^	RT 15% (2/13) CRT 12.5% (3/24)	NR	Neutropenia – CRT 8.3% (2/24)	NR
Hashimoto 2009^12^	RT 6.7% (1/15) CRT 0% (0/4)	NR	Neutropenia - RT 6.7% (1/ 15) CRT 25% (1/4) Anorexia – RT 13.3% (2/15) CRT 25% (1/4)	NR
Lee JA 2009^13^	0% (0/23)	NR	NR	NR
Asakura 2011^14^	RT 0% (0/18) CRT 0% (0/12)	NR	Neutropenia Grade 3 25% (3/12) CRT Grade 4 8.3% (1/12) CRT	GI hemorrhage Grade 3 8.3% (1/12) CRT
Chaw 2014^5^	NR	NR	NR	NR
Tey 2014^15^	0.9% (1/115)	Nil	Gastritis 0.9% (1/115) Anorexia 0.9% (1/115)	Nil
Kondoh 2015^16^	NR	Nil	Neutropenia- CRT 20%(3/15)	Nil

## DISCUSSION

To the best of our knowledge, this is the first systematic review that summarized quantitatively the results of studies with different dose fractionation schedules of palliative gastric RT for locally advanced GC. Despite a comprehensive literature search, we found only seven relevant studies reporting outcomes of palliative RT for GC. We did not find any prospective studies that complied with our search criteria. Our review showed that RT for localised GC symptoms had high response rates, with pooled overall response rates for bleeding, pain and obstruction symptoms were 74%, 67% and 68% respectively. This is consistent with palliative RT for other organ sites.[17, 18]

Whilst chemotherapy can improve the survival for patients with advanced disease, it may be inadequate for the palliation of local symptoms. RT alone can provide adequate palliation without the morbidity of chemotherapy. In addition, it may be the only option for elderly patients not fit for chemotherapy, patients with poor performance status, or patients who have progressed on chemotherapy.

Although there was significant heterogeneity in the dose fractionation regimens used (ranging from 8Gy to 50Gy in 1.8 to 8Gy per fraction), the pooled overall response rate of ≥67% suggests that RT is effective in palliating localised gastric bleeding/pain and obstruction. Pooled response for bleeding according to BED showed that there was no difference in response rates between regimens with BED of ≥ 39Gy versus regimens with BED<39Gy. Lack of a dose response relationship has also been demonstrated for palliative RT for other cancers. The Medical Research Council BA09 trial compared 2 hypofractionated regimens of RT in the palliation of muscle invasive bladder cancer, and concluded that the 21Gy/3 fraction regimen (low BED) appeared to be as effective as the 35Gy/10 fraction regimen (high BED) [18] In a systematic review of palliative thoracic RT for lung cancer, Fairchild et al also demonstrated that using a cut-off BED of 35Gy(α/β=10), there was no difference in palliation of hemoptysis, chest pain and cough between high dose (≥ 35Gy) vs. low dose (<35Gy) regimens.[17] These results, together with the results from our study suggest low BED regimens may be equally efficacious at symptom palliation compared to higher BED regimen. Short fractionation (low BED) regimens may be more desirable and preferred in patients with limited life expectancy, poor functional status, and who need to receive early systemic treatment for rapidly progressing systemic disease. In addition to symptom response, duration of palliation is an important endpoint for palliative treatments. Duration of symptom palliation was not reported in 3 of the 7 included studies. Two studies suggested that duration of palliation was inferior for RT regimens with BED of ≤41Gy, highlighting that prospective trials are needed to determine the optimal dose fractionation regimens for patients.

Validated grading scales such as RTOG or CTC were used in the studies for toxicity assessment. However, in retrospective studies, reviews of toxicity rates and duration cannot be accurately estimated. The absence of patient reported outcomes (PROs), such as quality of life assessments may make toxicity from treatment difficult to interpret. Nausea during or after treatment for example, may be due to various factors such as increasing doses of analgesia, tumour progression or may be treatment related. Validated questionnaires such as the European Organisation for Research and Treatment of Cancer (EORTC) Quality of life questionnaire C30 or the EORTC gastric cancer module should be used for PRO reporting.

Twenty percent of patients were treated with chemoradiotherapy. The results show that chemoradiotherapy is associated with increased toxicities with uncertain benefit on symptom palliation. With the exception of one study [16], symptom response was not reported according to whether the patient received RT alone or chemoradiotherapy. Toxicities appear to be acceptable for patients treated with RT alone. Grade 3 to 4 acute toxicities occurred in up to 15% of patients for patients treated with RT alone and up to 25% of patients treated with chemoradiotherapy. One study reported late grade 3 Gastrointestinal hemorrhage in 8% (1/12) patients who underwent chemoradiotherapy.[14] Indeed, the TROG 03.01,NCIC CTG ES2 randomised trial showed that palliation of dysphagia for patients with advanced esophageal cancer was not siginificantly improved with the addition of cisplatin/ 5 flurouracil chemotherapy to RT, but was associated with increased toxicity.[19] Further research is required to define the role of chemoradiotherapy in palliating localized gastric symptoms.

Whilst this review showed a benefit for palliative RT for localised GC symptoms, the reviewers also acknowledge that the included studies had several limitations. Firstly, a wide range of dose fractionation regimens were used with varying definitions of response to RT for bleeding, pain and obstruction, as well as time points for assessment of treatment response. This precludes the conclusions of dose response or most appropriate dose fractionation regimens. Secondly, twenty percent of patients were treated with chemoradiotherapy, which may influence response rates and treatment toxicity. Thirdly, no study reported PROs, which are important in the measures of effects of palliative treatment and should now be routinely included in studies of palliative interventions. Lastly, retrospective studies are at risk of reviewer bias, leading to overestimation of treatment effect or underestimation of treatment toxicities.

## CONCLUSIONs

This review suggests that two-thirds of patients receiving RT will experience a clinical benefit, with the highest response rate for haemostasis. Low BED regimens appear to be adequate for symptom palliation. Toxicity rates appear acceptable for patients treated with RT alone but are significanty increased with chemoradiotherapy.

The optimal dose fractionation regimen for symptom palliation is unclear. Prospective studies to determine the effects of palliative RT in inoperable gastric cancer on HRQL outcomes are warranted.

## MATERIALS AND METHODS

### Search strategy

Searches of the Medline and Central library databases were performed up till December 2015. The following MESH terms were used in the search strategy: Stomach neoplasm, radiotherapy, and palliative care. Results were screened by two authors. Full text copies of studies were obtained. Additional studies were identified from the reference lists of the articles reviewed in full text.

### Eligibility

We included studies of palliative gastric RT for patients with GC. Studies that reported symptom response (bleeding, pain, obstruction), toxicity or quality of life were included. We included retrospective reviews and non randomized studies. Case reports were excluded. Studies that included patients treated with RT as a subgroup was included as long as outcome of palliative RT for the subgroup was reported.

### Evaluation of studies

We adopted the Newcastle-Ottawa quality assessment scale for assessment of quality as we included non-comparative studies in our systematic review. [7] We selected items that were evaluated if: (1) The study patients were representative of the population of interest, (2) the outcome of interest was demonstrated to be absent at the start of the study, (3) there was adequate assessment of outcome, (4) there was sufficient length of follow up to allow outcomes to arise, and (5) there was adequacy of follow up (i.e. all patients in the study were accounted for) (Figure 3). Potential articles were evaluated by two authors and differences were resolved consensually.

Treatment parameters collected included RT planning (CT planned vs 2D planned), organ at risk dose constraints, RT dose fractionation regimen, RT technique. Outcomes of interest included symptom response according the the authors’ definition, duration of palliation, median survival, BED calculation and correlation, quality of life, acute and late treatment toxicities.

### Statistical analysis

We calculated event rates of outcome i.e. the proportion of patients who developed outcomes of interest from the included cohorts and estimated the 95% confidence interval with the Jeffreys method.[8] We combined the individual log-transformed event rates and their variances using the generic inverse variance method. The meta-analysis was performed using the Cochrane Collaboration software (RevMan version 5.2; http://www.cochrane.org). Primary analyses were done with Der Simonian and Laird random effects model.[9] Statistical heterogeneity of the combined results was assessed by the I2 statistic.[10] An I^2^ value of lower than 25% was interpreted as signifying a low level of heterogeneity. We used the Altman's test of interaction to compare the log-transformed rates of outcomes between the three treatment strategies.[11]

### Subgroup analysis

Subgroup analyses were performed to determine if the results were influenced by the BED. To analyse for a dose response relationship for patients who presented with bleeding, we used the cut-off BED of 39Gy, corresponding to the most commonly prescribed dose fractionation regimen of 30Gy in 10 fractions.

The BED is an approximate quantity by which different RT fractionation regimens are compared. It is given by BED = nD(1 + [D/{α/β}]), where n=number of fractions, D=dose/fraction, nD=total dose, and α/β is the alpha/beta ratio, and is taken to be 10 for adenocarcinomas.
